# Genome-Wide Characterization of the DELLA Gene Family in *Hevea brasiliensis* Reveals Evolutionary Diversification and Potential HbDELLA3/4–HbbHLH25 Interaction

**DOI:** 10.3390/biology15191691

**Published:** 2026-09-22

**Authors:** Xiaoling Tang, Jiale Liu, Songle Fan, Xiaoxiran Zhao, Mingyang Liu, Lifeng Wang

**Affiliations:** 1 State Key Laboratory of Tropical Crop Breeding, Sanya Institute of Breeding and Multiplication, School of Tropical Agriculture and Forestry, Hainan University, Sanya 572025, China; tangxlrri@163.com (X.T.); liujialehainanniu@163.com (J.L.); 2 Key Laboratory of Biology and Genetic Resources of Rubber Tree, Ministry of Agriculture and Rural Affairs/State Key Laboratory Incubation Base for Cultivation & Physiology of Tropical Crops, Rubber Research Institute, Chinese Academy of Tropical Agricultural Sciences, Haikou 571101, China; slfan@catas.cn (S.F.); liumy2023@126.com (M.L.); 3 College of Tropical Crops, Yunnan Agricultural University, Kunming, 650201, China; zhaoxiaoxiran@163.com

**Keywords:** DELLA, HbbHLH25, *Hevea brasiliensis*, gibberellin, phylogenetic analysis

## Abstract

DELLA proteins are central negative regulators of gibberellin signaling and play important roles in plant growth, development, and environmental responses. However, the DELLA gene family has not been systematically characterized in *Hevea brasiliensis*. Here, we identified members of the HbDELLA protein family in the rubber tree genome and examined their evolutionary relationships, conserved motifs, domain organization, chromosomal distribution, collinearity, expression patterns, and protein interactions using comparative genomics, transcript profiling, and yeast two-hybrid assays. Five HbDELLA proteins were identified. Comparative analysis of DELLA proteins from 14 representative species showed that family size ranged from one to five members and that DELLA proteins contain conserved C-terminal GRAS domains but divergent N-terminal DELLA-related regions. Phylogenetic analyses resolved two major clades, S1 and S2, with HbDELLA3 and HbDELLA4 clustering with AtGAI and AtRGA in subclade S1.1. Collinearity analysis suggested that WGD or segmental duplication contributed mainly to HbDELLA family expansion. *HbDELLA* genes showed tissue- and developmental-stage-specific expression and distinct temporal responses to gibberellin treatment in flowers. Yeast two-hybrid assays supported interactions of HbDELLA3 and HbDELLA4 with HbbHLH25 in yeast. These findings identify candidate protein associations for further investigation but do not establish their occurrence or regulatory functions in rubber tree cells.

## Simple Summary

DELLA proteins act as brakes that slow down plant growth when they are present. In rubber trees, the main source of natural rubber, scientists studied these proteins to understand how they work. Five DELLA proteins were found in the rubber tree genome. The researchers traced their evolutionary history and found that these proteins shared similar core structures but had some differences in their outer parts. Two specific proteins, called HbDELLA3 and HbDELLA4, could interact with a partner protein, HbbHLH25, which is located in the cell nucleus. This candidate interaction may help control how the plant grows and responds to gibberellin, a growth hormone. The scientists also showed that these DELLA proteins are active in different parts of the plant at different times. This study helps explain how rubber tree growth and development are regulated at the molecular level. Understanding this could lead to better ways for improving rubber yield and plant health, which is important for the natural rubber industry that produces materials used in tires, medical gloves, and many other everyday products.

## 1. Introduction

The rubber tree (*Hevea brasiliensis*) is the primary commercial source of natural rubber. Its latex yield and the growth and development status of the plant directly affect the stable development of the natural rubber industry [1,2]. In the rubber tree, a perennial woody economic crop, growth, flowering, laticifer differentiation, latex metabolism, and stress adaptation are all regulated by a complex endogenous signaling network [3,4,5]. Among them, plant hormones play an important role in coordinating vegetative growth, reproductive development, pest and disease resistance, and environmental responses [6,7,8]. Gibberellin (GA) is one of the important hormones that regulate plant height, cell elongation, seed germination, flowering, and vascular tissue development [9]. Research shows that exogenous application of GA can inhibit flowering in rubber trees [4].

In the GA signaling pathway, DELLA proteins act as the core negative regulatory elements of the pathway and play a key role in plant hormone signal transduction, growth and development, as well as stress resistance and disease resistance regulation [10,11]. DELLA proteins belong to the GAI-RGA-and-SCR (GRAS) family, and their protein structural features are highly conserved. The N-terminal DELLA and TVHYNP domains are key regions for recognition and binding by the GA receptor GID1. Mutations in these two domains can affect the protein’s ability to bind to the GA receptor GID1 [12,13]. The GRAS functional domain is located at the C-terminus and consists of two leucine-rich repeat sequences (LHR I and LHR II) and three conserved sequences: VHIID, PFYRE, and SAW [13]. The canonical function of DELLA can be summarized as follows: Under conditions of low gibberellin (GA) concentration, DELLA proteins accumulate in plant cells and exert an inhibitory effect on plant growth; under conditions of high GA concentration, GA binds to its receptor GID1, which in turn promotes the ubiquitination of DELLA and subsequent degradation via the 26S proteasome pathway, ultimately eliminating the inhibitory effect on plant growth [14,15]. Early studies mainly regarded DELLA as a negative regulator of the GA signaling pathway, explaining phenotypes such as dwarfism, internode elongation, seed germination, and flowering [15,16]. For example, the *Arabidopsis thaliana* gai mutant exhibits a dwarf phenotype and is insensitive to exogenous gibberellin (GA) treatment. This mutation blocks the conformational transition of GAI, a DELLA family protein, to the active conformation: after the GA receptor Gibberellin Insensitive Dwarf 1 (GID1) perceives and binds to bioactive GA, it interacts with DELLA proteins and induces conformational changes in DELLA proteins. The aforementioned mutation causes persistent accumulation of GAI in plant tissues, ultimately leading to the inhibition of plant growth [17]. In *Arabidopsis thaliana*, loss-of-function DELLA mutants were obtained through screening of suppressors for the extreme dwarf phenotype of the GA biosynthesis mutant *ga1-3*, and the corresponding locus was named RGA [18]. RGA and GAI were core inhibitors of vegetative growth and floral induction in *Arabidopsis thaliana* [19]. Furthermore, RGA and GAI exhibited partial functional redundancy in maintaining the repressive state of the GA signaling pathway, but the functional contribution of RGA was greater than that of GAI [20]. In contrast to the other four AtDELLA proteins, RGL2 played a predominant role in the regulation of seed germination [21,22]. Floral development was jointly regulated by RGA, RGL1 and RGL2. Due to the widespread functional redundancy among DELLA proteins, the loss of RGA function can only partially rescue the phenotypic defects of *ga1*; by contrast, the quintuple mutant with deletion of all five DELLA genes can completely restore all GA-deficient phenotypes of ga1 [23]. In GA signal transduction, the DELLA protein is a core negative regulatory factor and is designated SLR1 in rice. The accumulation of SLR1 protein inhibits GA signal transduction, while in the presence of GA, the GA receptor GID1 binds to the DELLA/TVHYNP domain at the N-terminus of SLR1, which triggers F-box protein-mediated ubiquitination and degradation of SLR1, thereby eliminating the inhibitory effect of SLR1 on GA signaling [24].

An increasing number of studies indicate that DELLA is a multi-signal integration platform capable of interacting with numerous transcription factors and transcriptional regulatory proteins, coordinating hormone, light, nutrient, stress, and developmental signals [11]. DELLA is referred to as the “Green Revolution gene,” emphasizing that it is not only a GA signaling repressor but also a central regulatory factor responding to endogenous hormones and external environmental signals [9]. Therefore, the core scientific issue in DELLA research is no longer confined to the mechanism by which GA induces DELLA degradation, but has transitioned to elucidating how DELLA mediates the trade-off between plant growth, defense response and reproductive development across different tissues and under various environmental conditions through protein–protein interactions, chromatin binding and post-translational modifications. Existing studies have demonstrated that DELLA can interact with hundreds of biological molecules, including transcription factors, transcriptional regulators, chromatin remodeling factors, chromatin modifying factors as well as histones [25]. Current research holds that DELLA functions as a transcriptional hub that integrates environmental signals and developmental cues into the overall transcriptional regulatory network.

Currently, the DELLA family has been identified and functionally studied in various plants such as Arabidopsis, rice, wheat, maize, and tomato [26,27,28,29,30], but systematic research in rubber trees remains relatively limited. As a perennial tropical woody latex-producing plant, the rubber tree has a long growth cycle and a complex genome. The composition, evolutionary relationships, chromosomal distribution, collinearity relationships, and expression patterns of DELLA family members in rubber trees are still unclear. Therefore, systematic genome-wide identification and evolutionary analysis of the rubber tree DELLA gene family will facilitate elucidation of the molecular mechanism underlying GA signaling regulation during rubber tree growth, development and latex biosynthesis-related biological processes.

In this study, a comparative evolutionary analysis of DELLA proteins across 14 representative plant species was performed. Investigation of protein physicochemical properties, conserved motifs, domain architecture, phylogenetic relationships, chromosomal distribution and collinearity clarified the sequence characteristics and evolutionary trajectories of the HbDELLA gene family in rubber tree. Furthermore, the expression profiles of HbDELLA genes were characterized based on transcriptomic data across multiple tissues and developmental stages. Yeast two-hybrid assays were subsequently conducted to detect the physical interaction between HbDELLA proteins and HbbHLH25. Combined with the results of subcellular localization assays of HbbHLH25, this study revealed that HbDELLA may participate in the crosstalk mechanism between GA signaling and the bHLH transcriptional regulatory network. The findings of this research provide a foundational framework for further functional dissection of the roles of DELLA-mediated GA signaling in rubber tree growth, development and latex biosynthesis-associated biological processes.

## 2. Materials and Methods

### 2.1. Genome-Wide Identification of DELLA

Genome sequences and annotation data for the 14 selected representative plant species were retrieved from the NCBI Genome Database (https://www.ncbi.nlm.nih.gov/genome) (accessed on 1 March 2025) and the National Genomics Data Center (https://www.ngdc.cncb.ac.cn/) (accessed on 5 March 2025). For genes with multiple alternatively spliced transcripts, only the longest transcript was retained for subsequent analysis after processing the genome annotation files, and the corresponding nucleotide and protein sequences were extracted. For *Hevea brasiliensis*, the haplotype A (hapA) assembly of the haplotype-resolved telomere-to-telomere genome of cultivar CATAS 73397 was used for genome-wide identification of HbDELLA genes. The hapA genome sequence and its corresponding gene annotation were obtained from the version 1 Figshare dataset (doi:10.6084/m9.figshare.26644396.v1; associated NGDC BioProject: PRJCA022207) (accessed on 5 March 2025). Full-length protein sequences of the five AtDELLA family members were downloaded from the TAIR database (https://www.arabidopsis.org/) (accessed on 5 March 2025). A combined strategy of HMMER search (based on the hidden Markov model corresponding to Pfam entry PF03514) and Blastp homology alignment (with an E-value threshold of 10^−5^) was implemented to identify DELLA family genes [31]. After the results from the two methods were combined, the Conserved Domain Database was used to filter out false-positive candidates, and only genes encoding proteins that simultaneously contained both the DELLA domain and the GRAS domain were retained as final DELLA family members [32]. The evolutionary relationship tree of 14 species was obtained through the online platform Timetree 5 (https://timetree.org/) (accessed on 10 March 2025).

### 2.2. DELLA Domains, MEME Cluster Analysis, and Collinearity Analysis

The MEME online platform (https://meme-suite.org/meme/tools/meme) (accessed on 20 March 2025) was used to perform motif analysis on 34 members of the DELLA family [33]. The Conserved Domain Database and the ProtParam online platform were used to analyze the domains and physicochemical properties of 34 members of the DELLA family [32]. Collinearity analysis among HbDELLA family members in rubber tree was performed using MCScanX software (https://github.com/wyp1125/MCScanX) (accessed on 25 March 2025), and the gene duplication types of the family members were classified [34]. Collinearity analysis of DELLA family members among rubber tree, *Arabidopsis thaliana*, and *Amborella trichopoda* was also conducted using MCScanX. Based on the GFF annotation file of the rubber tree genome, the chromosomal localization map of HbDELLA genes was constructed to visualize their distribution positions on the assembled chromosomes.

### 2.3. Construction of the DELLA Phylogenetic Tree

Phylogenetic analyses were performed separately using the full-length amino acid sequences, DELLA domain sequences, and GRAS domain sequences of the 34 identified DELLA proteins. Domain boundaries were determined based on Conserved Domain Database annotations. Multiple sequence alignments were generated using MUSCLE v5 (https://www.drive5.com/) (accessed on 25 March 2025) [35]. Maximum-likelihood phylogenetic trees were inferred using IQ-TREE 2 (https://iqtree.github.io/) (accessed on 25 March 2025) [36], and branch support was assessed with 1000 bootstrap replicates. The phylogenetic tree of DELLA family members was visualized and formatted using FigTree v1.4.4 (https://tree.bio.ed.ac.uk/software/figtree/) (accessed on 5 April 2025). 

### 2.4. Analysis of the Expression Patterns of HbDELLA in Different Tissues and Under Gibberellin Treatment

Previously published RNA-seq datasets were reanalyzed to characterize HbDELLA expression. Raw RNA-seq data from different tissues of *H. brasiliensis* were downloaded from the NCBI Sequence Read Archive under accession numbers SRR3136158, SRR3136165, SRR3136162, SRR3136159, SRR3136166, SRR3136156, and SRR3136168 and reanalyzed to characterize tissue-specific expression patterns of HbDELLA genes. The leaf developmental dataset comprised four sequencing runs deposited in the NCBI Sequence Read Archive. Samples were collected from 10-year-old *H. brasiliensis* trees of cultivar CATAS73397 at four developmental stages: bronze (SRR3136185), color-change (SRR3136188), pale-green (SRR3136190), and bright-green mature (SRR3136192) [37]. RNA-seq data utilized in the GA_3_ treatment analysis was generated by our research team in earlier work [4]. The original sequencing reads were obtained directly from the team and reanalyzed in the present study, rather than downloaded from a public repository. These data have not yet been deposited in a public database, and no public accession number is currently available. Inflorescences of cultivar CATAS73397, selected for uniform development, were sprayed with 1.5% GA_3_ or 0.05% Tween-20 as the control, with 5 mL applied per inflorescence. RNA-seq samples were collected at 0 (CK), 6, 12, 24, and 48 h after treatment, and 3 biological replicates per treatment and time point were included in the present analysis. Sequencing quality control and raw data filtering were performed using FastQC (https://www.bioinformatics.babraham.ac.uk/projects/fastqc/) (accessed on 5 April 2025) and Trimmomatic software (https://github.com/usadellab/Trimmomatic/) (accessed on 15 April 2025), respectively, to obtain high-quality clean reads [38,39]. The filtered clean reads were aligned to the recently released rubber tree reference genome [40] using HISAT2 software (https://ccb.jhu.edu/software/hisat/index.shtml) (accessed on 15 April 2025) [41]. Subsequently, transcriptome assembly and gene expression quantification were performed with StringTie software (https://ccb.jhu.edu/software/stringtie/) (accessed on 15 April 2025) [42]. Based on the obtained quantitative results of transcriptome data, the expression abundance of five HbDELLA family members was extracted, and the corresponding expression heatmap was constructed. Differential gene expression analysis was performed using DESeq2 (https://bioconductor.org/packages//release/bioc/html/DESeq2.html) (accessed on 15 April 2025) based on raw gene read counts.

### 2.5. Yeast Two-Hybrid Assays

For the HbbHLH25, HbDELLA1, HbDELLA2, HbDELLA3, and HbDELLA4 genes, specific primers containing homologous arms and the target coding sequences (excluding the stop codon) were designed (Table S2), and target fragments were obtained via PCR amplification. Using homologous recombination technology [43], the HbbHLH25 coding sequence was inserted into the pGBKT7 vector to construct the bait recombinant vector pGBKT7-HbbHLH25; meanwhile, each HbDELLA coding sequence was inserted into the corresponding pGADT7 prey vector to obtain recombinant plasmids pGADT7-HbDELLA1, pGADT7-HbDELLA2, pGADT7-HbDELLA3, and pGADT7-HbDELLA4, respectively. HbDELLA5 was not tested because its full-length coding sequence could not be obtained by cloning. Yeast two-hybrid assays were performed using the Y2HGold-GAL4 interaction validation kit (Coolaber, China; catalog no. YH2011-10T) according to the manufacturer’s instructions. The pGBKT7-HbbHLH25 bait construct and each pGADT7-HbDELLA1-4 prey construct were co-transformed into *Saccharomyces cerevisiae* strain Y2HGold using the PEG/LiAc method. Cells were collected by centrifugation, washed with sterile water, and plated on SD/−Leu/−Trp medium. Co-transformants were selected after incubation at 28–30 °C for 48–96 h.

For interaction assays, fresh colonies were resuspended in sterile water and adjusted to an OD600 of 0.2. Tenfold serial dilutions were prepared to obtain OD_600_ values of 0.2, 0.02, 0.002, and 0.0002. Aliquots of 10 μL were spotted onto SD/−Leu/−Trp and SD/−Leu/−Trp/−His/−Ade media and incubated at 28–30 °C for 3–5 days before growth was recorded. BD–HbbHLH25 paired with empty AD and each AD–HbDELLA construct paired with empty BD served as controls for background reporter activation. The pGBKT7-53/pGADT7-T and pGBKT7-Lam/pGADT7-T combinations served as positive and negative controls, respectively.

### 2.6. Subcellular Localization of HbbHLH25

Primers for HbbHLH25 were designed as follows: forward primer HbbHLH25-pGREEN-HindIII-F: gtcgacggtatcgataagcttATGCAAGAACCAAAGACCAAG; reverse primer HbbHLH25-pGREEN-BamHI-R: tttactcatactagtggatccTAGACTTTGGAGGAGGGCTCTTC. The HbbHLH25 gene was cloned by PCR, and the 35S: HbbHLH25: mGFP fusion expression vector was constructed using homologous recombination. Agrobacterium GV3101 was transformed with the construct. The infiltration solution was prepared, and tobacco leaf infiltration was performed as previously described [44]. The experimental group consisted of a mixture of *Agrobacterium* carrying 35S-HbbHLH25-GFP and *Agrobacterium* carrying an RFP membrane and nuclear localization marker. *Nicotiana benthamiana* plants were grown at 25 °C, and fully expanded leaves of 4-week-old plants were used for transient expression. Agrobacterium tumefaciens GV3101 cells carrying the 35S:HbbHLH25–mGFP construct or the RFP marker construct were harvested and resuspended in infiltration buffer containing 10 mM MgCl_2_, 10 mM MES (pH 5.6), and 150 μM acetosyringone. The suspensions were adjusted to an OD600 of 0.5. The mixture was infiltrated into the abaxial side of the leaves using a needleless syringe. Fluorescence was examined at 60 h post-infiltration using a confocal microscope (Leica, Wetzlar, Germany).

## 3. Results

### 3.1. Identification and Sequence Analysis of DELLA Family Members

To investigate the evolutionary distribution characteristics of the DELLA gene family across plants, the present study identified DELLA family members from the genomes of 14 representative plant species. The sampled taxa included bryophytes, lycophytes, gymnosperms, and angiosperms, with the latter represented by Amborella trichopoda, magnoliids, monocots, and eudicots. The results revealed that the number of DELLA family members varied among different plant species, ranging from 1 to 5 copies per genome (Figure 1A). One DELLA member was identified in each of the two sampled bryophytes, *Marchantia polymorpha* and *Physcomitrium patens*, and in the lycophyte *Selaginella moellendorffii*. The two sampled monocots, *Oryza sativa* and *Brachypodium distachyon*, also each contained one DELLA member. Among the remaining sampled species, *Ginkgo biloba* contained three members, *Amborella trichopoda* two, *Cinnamomum kanehirae* three, *Populus trichocarpa* four, *Arabidopsis thaliana* five, *Jatropha curcas* three, *Manihot esculenta* two, *Vernicia fordii* two, and *H. brasiliensis* five.

Analysis of MEME motifs and conserved domain annotation indicated that plant DELLA proteins generally exhibited conserved motif composition and the typical DELLA-GRAS domain architecture (Figure 1B). Specifically, the C-terminal GRAS domain was highly conserved across all analyzed taxa, whereas the N-terminal DELLA-specific region exhibited some sequence variation among different plant lineages. This finding suggested that despite the maintenance of conserved core functional structures in the DELLA family, a certain degree of structural variation may have accumulated during the differentiation of plant lineages.

Analysis of physicochemical properties showed that the length of DELLA proteins differed substantially among taxa, with the number of amino acid residues ranging from 481 to 731 aa (Table S1). Among the identified proteins, SmDELLA was the shortest (481 aa), while GbDELLA2 was the longest (731 aa). Correspondingly, the molecular weights of DELLA proteins ranged from 53.40 kDa to 79.96 kDa, consistent with the length variation: SmDELLA had the lowest molecular weight (53.40 kDa), and GbDELLA2 had the highest (79.96 kDa). Overall, most DELLA proteins had lengths between 530 and 640 aa and molecular weights between 58 and 70 kDa. This indicates that DELLA family proteins maintain a certain degree of length conservation across diverse plant species, though some gymnosperm DELLA members have undergone notable sequence elongation.

In rubber tree, the five HbDELLA proteins have amino acid lengths ranging from 538 to 636 aa, molecular weights ranging from 58.88 to 69.95 kDa, theoretical isoelectric points ranging from 5.19 to 5.45, instability indices ranging from 45.06 to 50.01, and GRAVY values ranging from −0.284 to −0.079 (Table S1). Compared with DELLA members from other plant species, the amino acid length, molecular weight, and theoretical pI of rubber tree DELLA proteins all fall within the typical range of the DELLA family, indicating that their basic physicochemical properties are relatively conserved evolutionarily.

### 3.2. Phylogenetic Relationships of DELLA Proteins

The phylogenetic analysis based on full-length DELLA protein sequences included the bryophyte proteins MpDELLA and PpDELLA, the lycophyte protein SmDELLA, and DELLA proteins from the sampled seed plants (Figure 2A). The identification of DELLA homologues in these taxa supports the distribution of this gene family across multiple land plant lineages. The three members of the gymnosperm Ginkgo biloba did not all cluster in the same branch, suggesting that the GbDELLA members may have undergone earlier duplication and differentiation. Angiosperm DELLA members were mainly distributed in the S1 branch, among which AtGAI and AtRGA clustered in subclade S1.1, while AtRGL1, AtRGL2, and AtRGL3 clustered in subclade S1.2, indicating that AtDELLA proteins can be clearly divided into two categories: GAI/RGA-like and RGL-like (Figure 2A).

The topology of the phylogenetic tree constructed based on the DELLA domain was generally consistent with that of the tree inferred from full-length sequences, which also divided DELLA members into two major branches, S1 and S2 (Figure 2B). S1.1 contains AtGAI, AtRGA, as well as some DELLA members from Populus trichocarpa, Manihot esculenta, Vernicia fordii, Jatropha curcas and *H. brasiliensis*; S1.2 harbors members including AtRGL1, AtRGL2, AtRGL3, PtDELLA3, PtDELLA4, JcDELLA1, HbDELLA1 and HbDELLA2. These results demonstrated that the DELLA domain can effectively distinguish between GAI/RGA-like and RGL-like DELLA members. However, some members exhibited different phylogenetic positions compared with those in the full-length sequence tree, indicating that the N-terminal DELLA region had accumulated a certain degree of sequence variation among different plant lineages (Figure 2B).

The phylogenetic tree constructed based on the GRAS domain also supported the classification of the DELLA family into two primary branches, S1 and S2 (Figure 2C). In comparison with the two aforementioned phylogenetic trees, the topological clustering relationships of several clades in the GRAS domain-based tree exhibited higher resolution. For instance, the monocotyledonous proteins OsDELLA and BdDELLA formed a tight monophyletic cluster, which indicated that the GRAS domains of DELLA proteins in the Poaceae were relatively conserved evolutionarily. Concurrently, AtGAI/AtRGA and AtRGL1/2/3 remained in subclades S1.1 and S1.2, respectively, which further corroborated the presence of stable subclade differentiation within the DELLA family. The five DELLA members from the rubber tree did not form a single monophyletic cluster, but were instead distributed across distinct subclades. Specifically, HbDELLA3 and HbDELLA4 exhibited the closest phylogenetic affinity to subclade S1.1, which harbors AtGAI/AtRGA; HbDELLA1 and HbDELLA2 showed the closest phylogenetic relationship to subclade S1.2, which contains AtRGL1/2/3, while HbDELLA5 clustered in clade S2 in all three phylogenetic trees, and showed a close phylogenetic relationship with members including JcDELLA3 and VfDELLA2. These results suggested that DELLA family members in the rubber tree may have originated from different ancestral copies, and had experienced functional differentiation during the evolutionary process.

### 3.3. Chromosomal Localization and Collinearity Analysis of Rubber Tree HbDELLA

The results demonstrated that the five HbDELLA genes were unevenly distributed across five distinct chromosomes, and were individually located on Chr9, Chr13, Chr15, Chr16 and Chr18, respectively (Figure 3A). Collinearity analysis revealed that there were significant collinear relationships among some HbDELLA members, which mainly involved HbDELLA1, HbDELLA2, HbDELLA3 and HbDELLA4 (Figure 3A). In addition, with the exception of HbDELLA5, the other HbDELLA members in rubber tree were derived from WGD (whole-genome duplication) or segmental duplication, suggesting that the expansion of the DELLA family in rubber tree was primarily associated with WGD or segmental duplication (Figure 3A). There were collinear relationships of varying degrees between the DELLA family members of rubber tree and those of Amborella trichopoda and *Arabidopsis thaliana* (Figure 3B). Specifically, HbDELLA3 and HbDELLA4 showed collinearity with AmtDELLA2 from *Amborella trichopoda*, supporting the conservation of the corresponding genomic regions between these species. Meanwhile, HbDELLA1, HbDELLA2, HbDELLA3 and HbDELLA4 exhibited collinearity with multiple DELLA members in Arabidopsis, suggesting that these genes were relatively conserved evolutionarily in eudicots.

### 3.4. Expression Patterns of HbDELLA in Different Tissues of the Rubber Tree and at Different Developmental Stages of Leaves

Across different tissues, HbDELLA1 exhibited the highest expression abundance and the broadest expression profile, with particularly high expression detected in female flowers, leaves and male flowers (Figure 4A). HbDELLA2 reached the highest expression level in latex. Both HbDELLA3 and HbDELLA4 displayed relatively high expression in female flowers; in addition, HbDELLA4 also showed high expression in leaves, whereas the expression of HbDELLA3 remained relatively low in other tissues (Figure 4A). Overall, HbDELLA5 was expressed at low levels in all tested tissues, with a certain number of transcripts only detected in female flowers and bark, and almost no expression detected in latex and seeds (Figure 4A). The HbDELLA family members exhibited higher transcript abundance in the bronze, color change and pale green leaf developmental stages, while the overall expression level decreased in the mature stage (Figure 4B). To investigate the regulatory effect of exogenous GA treatment on the expression of HbDELLA genes in rubber tree flowers, this study compared the expression levels of five HbDELLA members in the control (CK) and GA_3_ treatment groups at 6 h, 12 h, 24 h and 48 h post-treatment. The results indicated that different HbDELLA genes displayed significantly divergent response patterns to gibberellin treatment, exhibiting clear member-specific and time-specific expression characteristics (Figure 4C).

### 3.5. HbDELLA3/4 Both Interact with HbbHLH25, and HbbHLH25 Is Localized in the Nucleus

Among the experimental combinations, the strains co-transformed with HbbHLH25 + HbDELLA3 and HbbHLH25 + HbDELLA4 could grow normally on T-L-H-A quadruple dropout medium, while no obvious growth was detected for the HbbHLH25 + HbDELLA1 and HbbHLH25 + HbDELLA2 combinations. Yeast co-transformants expressing BD–HbbHLH25 with AD–HbDELLA3 or AD–HbDELLA4 grew on quadruple-dropout medium, whereas the corresponding empty-vector controls did not. These results support interactions between these protein pairs in the yeast assay (Figure 5). HbbHLH25–GFP showed predominantly nuclear localization in tobacco epidermal cells (Figure 6).

## 4. Discussion

DELLA proteins are core negative regulators of the plant gibberellin signaling pathway, and participate in multiple biological processes including plant growth and development, flowering regulation, seed germination, stress response, and crosstalk among multiple hormones signaling pathways [10,11]. In the present study, DELLA family members were identified in all 14 sampled land plant genomes, with family sizes ranging from one to five members (Figure 1A). One member was identified in each of the sampled bryophytes, Marchantia polymorpha and Physcomitrium patens, the lycophyte Selaginella moellendorffii, and the two monocots, Oryza sativa and Brachypodium distachyon. The remaining sampled species contained two to five members, with *Arabidopsis thaliana* and *H. brasiliensis* having the largest families in this dataset. These observations document variation in DELLA family size among the sampled species but do not, by themselves, establish the ancestral copy number or the timing of gene duplication and loss. Further analyses of conserved motifs and structural domains demonstrate that DELLA proteins across plant lineages generally retain high structural conservation (Figure 1B). All identified DELLA members possessed the canonical C-terminal GRAS domain, confirming that this domain constitutes a core structural foundation for maintaining the fundamental biological functions of DELLA proteins. In contrast, the N-terminal DELLA-associated region exhibited notable heterogeneity among different plant clades, which suggested that DELLA proteins had accumulated sequence variations of varying degrees during evolution while retaining their core structural and functional characteristics. This pattern of coexistence between structural conservation and sequence variation may provide a molecular foundation for DELLA proteins to participate in a diverse array of regulatory processes across different plant lineages.

Phylogenetic analyses based on full-length DELLA protein sequences and the DELLA and GRAS domains revealed relationships among DELLA homologues from the sampled bryophytes, lycophyte, and seed plants (Figure 2). All three phylogenetic trees divided the DELLA members into two main branches, S1 and S2, with S1 representing the major expansion branch of angiosperm DELLA members and further subdivided into two subbranches, S1.1 and S1.2. According to the clustering relationships of Arabidopsis DELLA members, S1.1 mainly corresponds to GAI/RGA-like members, while S1.2 mainly corresponds to RGL-like members. The five HbDELLA members in the rubber tree were distributed across S1.1, S1.2, and S2, with HbDELLA3 and HbDELLA4 located in S1.1, HbDELLA1 and HbDELLA2 in S1.2, and HbDELLA5 in the S2 clade. This result suggested that the DELLA family members in the rubber tree may have originated from different ancestral copies and undergone subfunctionalization during evolution. Chromosomal localization results showed that the five HbDELLA genes were distributed on different chromosomes, with no obvious tandem arrangement observed, indicating that tandem duplication may not be the main mode of expansion for the HbDELLA family (Figure 3A). Further collinearity analysis revealed collinearity relationships among some HbDELLA members as well as between HbDELLA members and DELLA members from other species, suggesting that WGD or segmental duplication may have contributed to the expansion of the DELLA family in the rubber tree (Figure 3). Previous studies had shown that gene duplication was an important driving force for adaptive evolution and the formation of complex traits in angiosperms, and WGD events had been frequently retained during angiosperm evolution [45]. Based on the results of this study, it was speculated that the expansion of the DELLA family in rubber trees was mainly related to WGD or segmental duplication, and different duplicated members may have undergone further functional differentiation during subsequent evolution.

This study revealed that distinct HbDELLA genes showed significant differences in their transcriptional responses to GA_3_ treatment, exhibiting both member-specific and time-specific expression patterns (Figure 4). Specifically, HbDELLA1 showed the highest transcript abundance in rubber tree flowers, and its expression was upregulated at 6 h and 48 h after GA_3_ treatment, indicating that HbDELLA1 may act as a functional member mediating GA signal responses in rubber tree flowers. HbDELLA3 and HbDELLA4 also exhibited clear GA-responsive expression characteristics. Considering their phylogenetic positions and collinearity relationships, it can be inferred that these two genes were relatively conserved GAI/RGA-like DELLA members in rubber trees and were involved in GA-regulated flower development and other relevant physiological processes. In contrast, HbDELLA5 maintained a relatively low overall expression level and clustered in the S2 clade of the phylogenetic tree, suggesting that its function may have diverged from that of other HbDELLA members.

In recent years, the understanding of DELLA protein function has evolved from its recognition solely as a negative regulator of GA signaling to its identification as a core hub that integrates multiple intracellular and extracellular signaling pathways. A growing body of research demonstrates that DELLA proteins can physically interact with a variety of transcription factors and regulatory proteins, thereby participating in the regulation of a wide range of biological processes including light signaling, hormone crosstalk, flowering development, abiotic and biotic stress responses, as well as plant growth and morphogenesis [25]. Specifically, DELLA proteins interact with diverse transcription factors to modulate their transcriptional activity and downstream biological metabolic processes. In particular, the interaction between DELLA proteins and bHLH-type transcription factors has been experimentally verified across multiple plant species. Moreover, PtRGL2 can directly bind to FT1/FT2 (FLOWERING LOCUS T) proteins via its GRAS domain, and negatively regulate the expression of FT1 and FT2, consequently promoting short-day-induced dormancy and suppressing low-temperature-mediated bud break [46]. The RGL1-ARF7-IAA9 module mediated the crosstalk between auxin and GA signaling pathways, which plays a critical role in the precise regulation of cambium development in Populus [47]. DELLA proteins can interact with the functionally redundant floral repressors IDD14/15/16 in *Arabidopsis thaliana*, acting as co-repressors to significantly enhance the transcriptional repression activity of these proteins on FT [48]. Results from yeast two-hybrid assays demonstrated that AtRGA can interact with six members of the bHLH transcription factor family, namely FT, ORG3, MYC2, PIF5, UNE10 and SACL1 [25]. AtRGL interacted with bHLH48 and bHLH60 proteins to suppress their transcriptional activation activity, and thereby regulated the GA-mediated flowering process in plants [49]. In addition, DELLAs can interact with PIF1 and PIF5, two members of the bHLH family, and promote the degradation of these PIF proteins via the ubiquitin-proteasome pathway [50]. BBX24 can physically interact with DELLA proteins, and attenuate the inhibitory effect of DELLA on PIF4 activity. Both OsbHLH089 and OsbHLH094 interacted with the rice DELLA protein OsSLR1 to participate in the regulation of pollen and stamen development [51]. Collectively, existing studies suggest that the DELLA–bHLH interaction module may serve as a key node connecting plant GA signaling to the downstream transcriptional regulatory network.

In this study, yeast two-hybrid assays were further performed to confirm that both HbDELLA3 and HbDELLA4 can interact with HbbHLH25 in yeast cells; in contrast, the interactions between HbDELLA1/HbDELLA2 and HbbHLH25 were undetectable. Phylogenetic and collinearity analyses showed that HbDELLA3 and HbDELLA4 clustered with AtGAI and AtRGA in subclade S1.1 and exhibited collinearity with AmtDELLA2 from Amborella trichopoda and with AtGAI/AtRGA from *Arabidopsis thaliana*. Together, these results support the evolutionary conservation of these DELLA homologues across the sampled angiosperm species. Meanwhile, subcellular localization analysis confirmed that HbbHLH25 localized to the nucleus, which was consistent with the fundamental characteristics of bHLH transcription factors. Overexpression of IbbHLH25 can significantly elevate carotenoid content in storage roots of sweet potato [52]. OsbHLH25 directly mediates hydrogen peroxide perception to enhance plant resistance against various fungal and bacterial pathogens [53]. Phylogenetic analysis showed that HbbHLH25 clustered directly with pumpkin disease resistance protein CmbHLH18, and belonged to subfamily IV together with rice disease resistance protein OsbHLH25 and apple disease resistance protein MdbHLH92 [54]. The yeast two-hybrid results identify HbDELLA3 and HbDELLA4 as candidate interacting partners of HbbHLH25. Although these observations motivate the hypothesis that HbbHLH25 may participate in DELLA-associated regulation, the interactions have not been independently validated. The nuclear localization of HbbHLH25 is compatible with such a hypothesis but does not demonstrate its association with HbDELLA proteins in plant cells. Moreover, the present data do not establish whether these associations affect HbbHLH25 activity, GA responses, or rubber tree development. Independent interaction assays and functional analyses are therefore required to determine their biological relevance.

## 5. Conclusions

This study identified five HbDELLA genes in the hapA genome assembly of *H. brasiliensis* cultivar CATAS 7-33-97 and characterized their sequence features, phylogenetic relationships, genomic distribution, and expression patterns. Duplication and collinearity analyses suggested that whole-genome or segmental duplication may have contributed to HbDELLA family expansion. HbDELLA genes exhibited variation in expression across tissues, leaf developmental stages, and GA_3_ treatments. Yeast two-hybrid assays supported interactions of HbDELLA3 and HbDELLA4 with HbbHLH25 in yeast. These findings provide a basis for further functional investigation, while the interactions in plant cells and their physiological significance remain to be established.

## Figures and Tables

**Figure 1:**
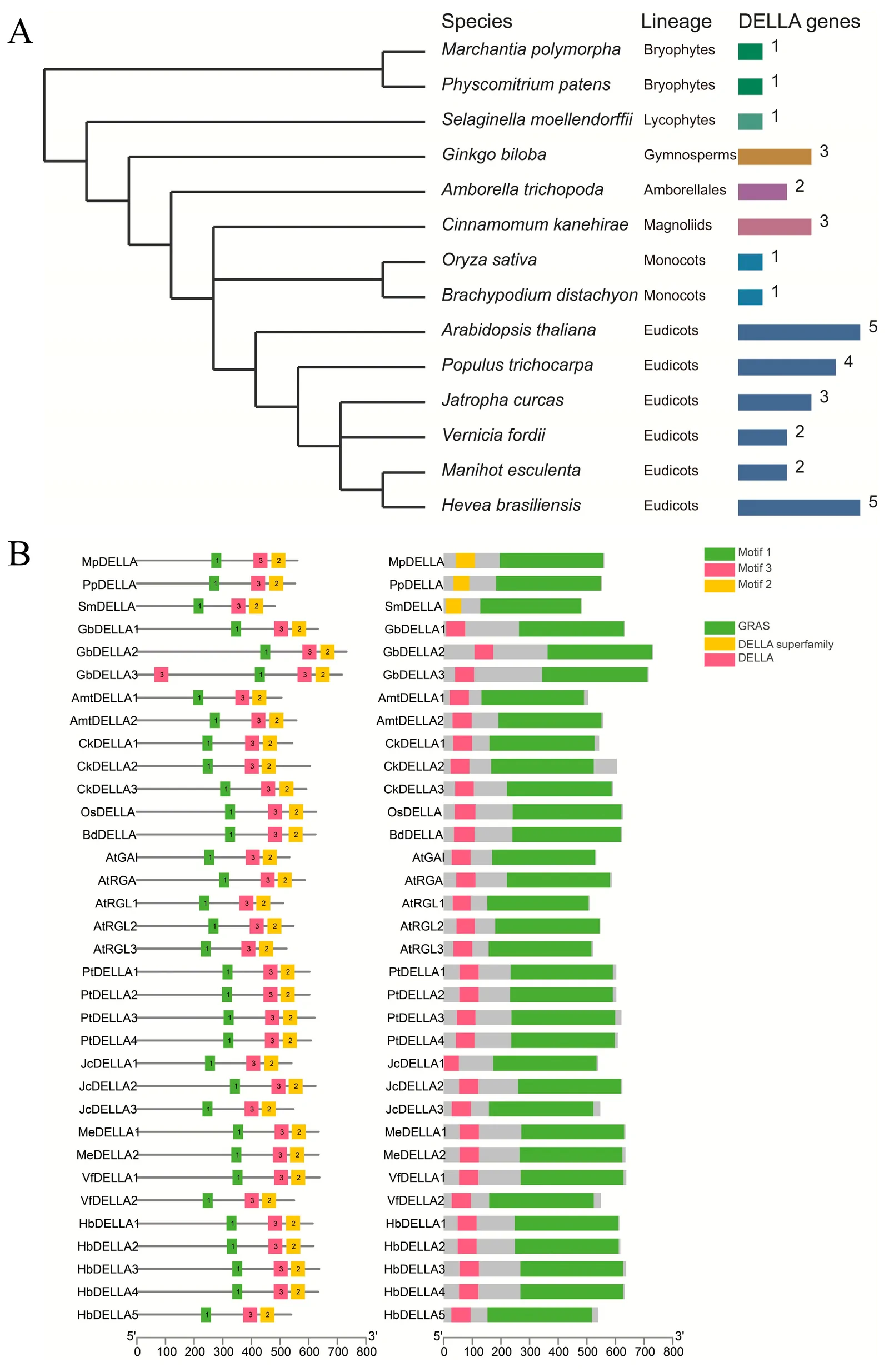
Distribution, conserved motifs, and domain organization of DELLA family members across 14 representative land plant species. (**A**) Schematic species tree and the number of DELLA genes identified in each genome. The tree summarizes published phylogenetic relationships and includes only the sampled taxa. Branch lengths do not represent divergence times or evolutionary distances; multifurcations indicate relationships left unresolved in this schematic. (**B**) Analysis of conserved motifs and domains of DELLA proteins in different plants. The left side shows the distribution of conserved motifs identified by MEME, while the right side displays the domain composition of DELLA proteins. Different colors represent different motifs or domains. Overall, DELLA proteins exhibit a relatively conserved motif arrangement pattern and contain a typical GRAS domain at the C-terminus.

**Figure 2:**
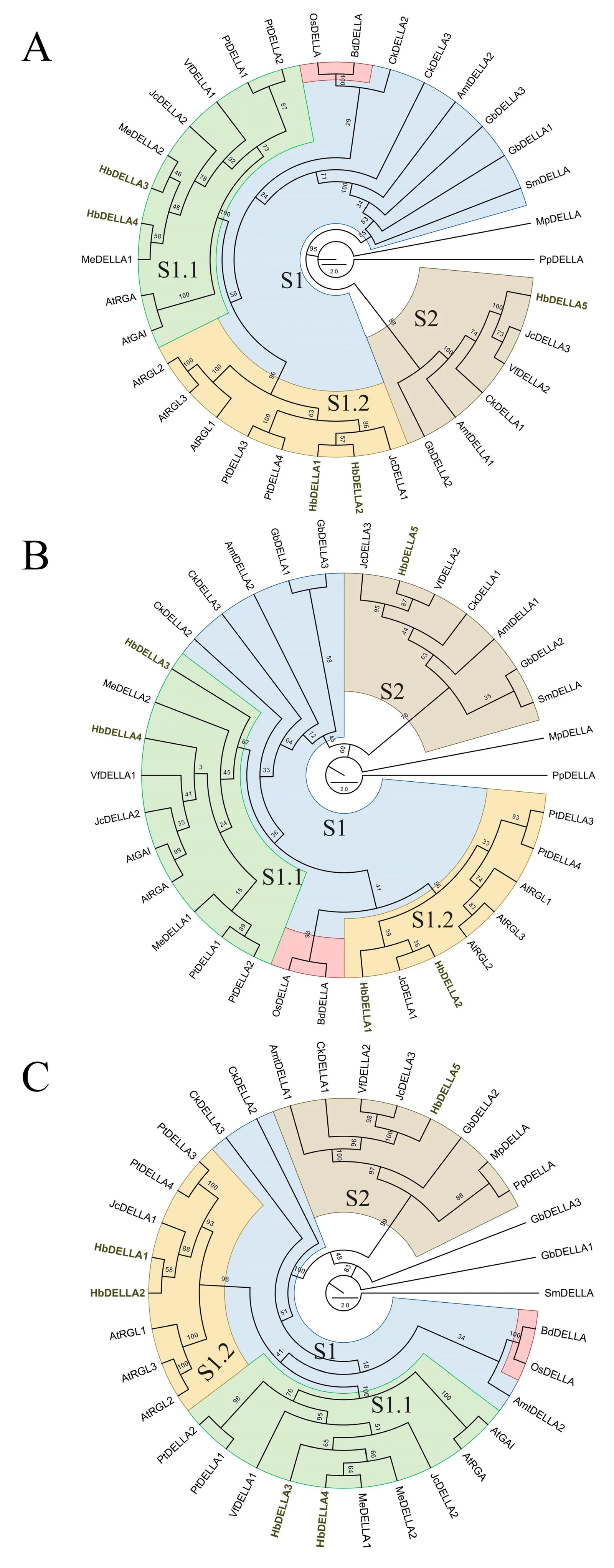
Phylogenetic trees constructed based on the full-length DELLA protein sequences, the DELLA domain, and the GRAS domain. (**A**) Phylogenetic tree constructed based on the full-length DELLA protein sequences. (**B**) Phylogenetic tree constructed based on the DELLA domain. (**C**) Phylogenetic tree constructed based on the GRAS domain. Different colors represent different evolutionary branches. All three phylogenetic trees divide the DELLA proteins into two main branches, S1 and S2, with S1 further subdivided into two sub-branches, S1.1 and S1.2.

**Figure 3:**
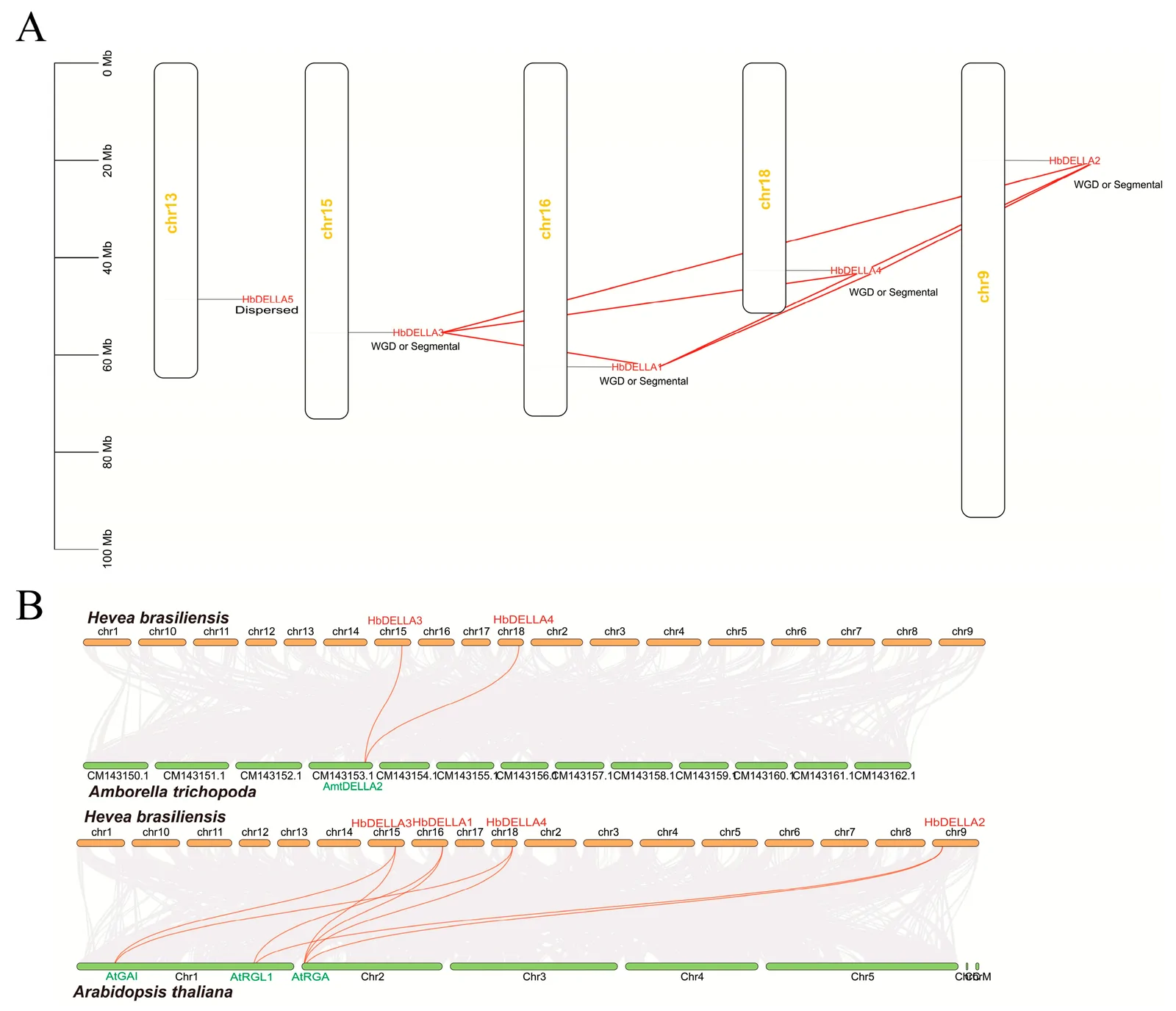
Chromosomal localization and collinearity analysis of HbDELLA genes in rubber tree. (**A**) Chromosomal distribution of HbDELLA genes in the rubber tree genome and collinearity among members. (**B**) Collinearity analysis of *H. brasiliensis* DELLA *genes* with DELLA genes from *Amborella trichopoda* and *Arabidopsis thaliana*.

**Figure 4:**
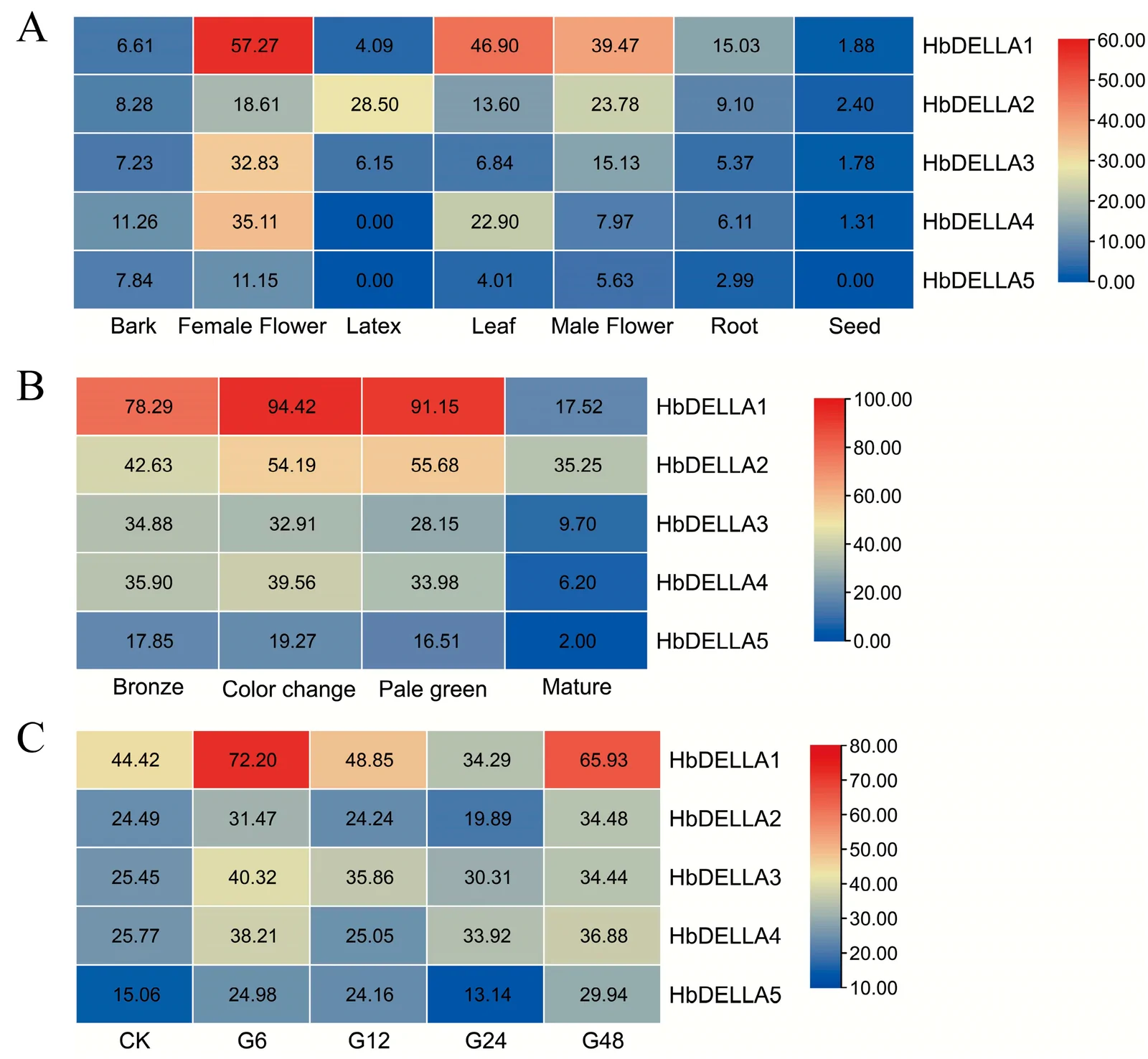
Expression pattern analysis of *HbDELLA* genes in rubber tree across different tissues and developmental stages and under gibberellin treatment. (**A**) Expression profiles of *HbDELLA* genes in different tissues. (**B**) Expression profiles of *HbDELLA* genes across four leaf developmental stages in rubber tree, following the staging system: I, bronze young leaves functioning as sinks; II, color-change leaves functioning as sinks; III, pale-green leaves undergoing the sink-to-source transition; and IV, bright-green mature leaves functioning as sources. These species-specific stages describe vascular leaf development (Plant Ontology, PO:0025570), with mature stage IV corresponding to the vascular leaf post-expansion stage (PO:0001053). (**C**) Expression profiles of *HbDELLA* genes in rubber tree flowers at different time points after gibberellin treatment. Samples were collected at 0, 6, 12, 24, and 48 h after treatment. The source study did not specify a formal developmental stage for the inflorescences at treatment initiation. The color gradient from blue to red corresponds to an increase in gene expression level from low to high.

**Figure 5:**
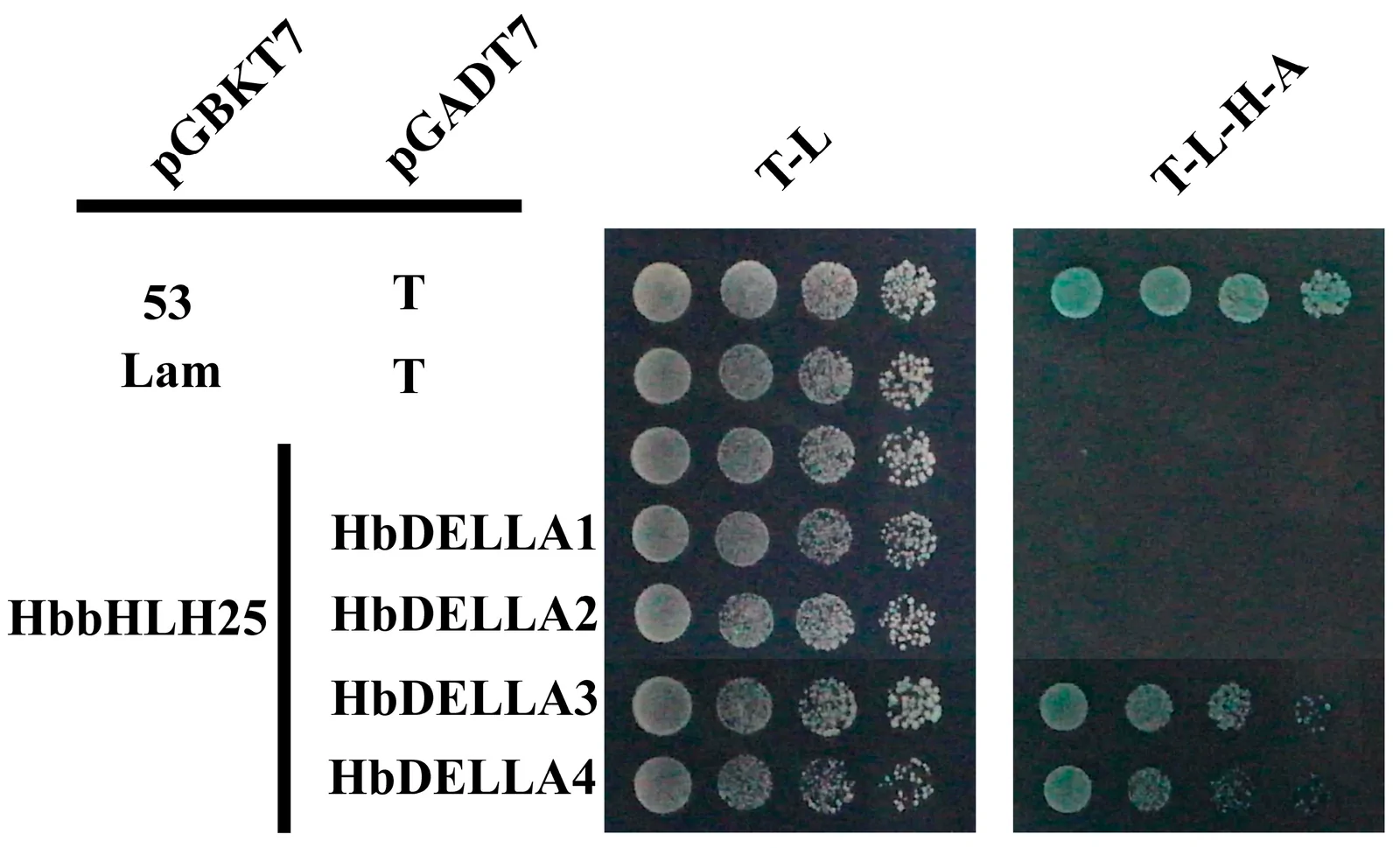
Yeast two-hybrid analysis of interactions between HbbHLH25 and HbDELLA proteins. pGBKT7-53/pGADT7-T and pGBKT7-Lam/pGADT7-T served as positive and negative controls, respectively.

**Figure 6:**
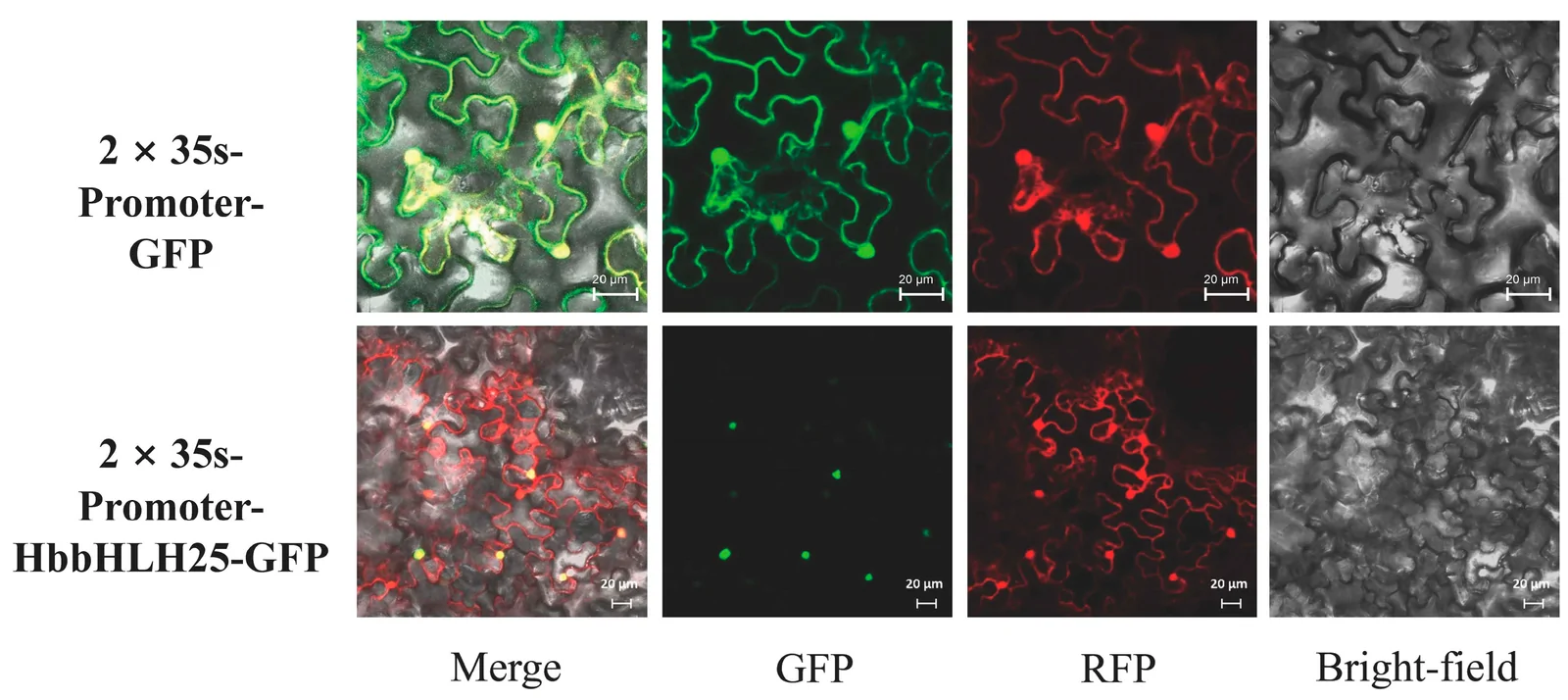
Subcellular localization assay of HbbHLH25 protein in tobacco leaf epidermal cells. RFP was used as the marker for subcellular localization at the plasma membrane and nucleus. From left to right, the panels sequentially present the merged image of GFP and RFP fluorescence, the GFP fluorescence signal, the RFP fluorescence signal, and the bright-field micrograph.

## Data Availability

The original contributions presented in this study are included in the article/Supplementary Materials. Further inquiries can be directed to the corresponding author.

## Supplementary Materials

The following supporting information can be downloaded at https://www.mdpi.com/article/10.3390/biology15191691/s1, Table S1: Physicochemical properties of DELLA family proteins in representative plant species. Table S2: List of primers used in the yeast two-hybrid experiment.
